# Supplementary figures and images for: Transgenerational inheritance of centromere identity requires the CENP-A N-terminal tail in the C. elegans maternal germ line
Source: PLoS Biol. 2021 Jul 6;19(7):e3000968. doi: 10.1371/journal.pbio.3000968 (PMC8259991; doi:10.1371/journal.pbio.3000968)

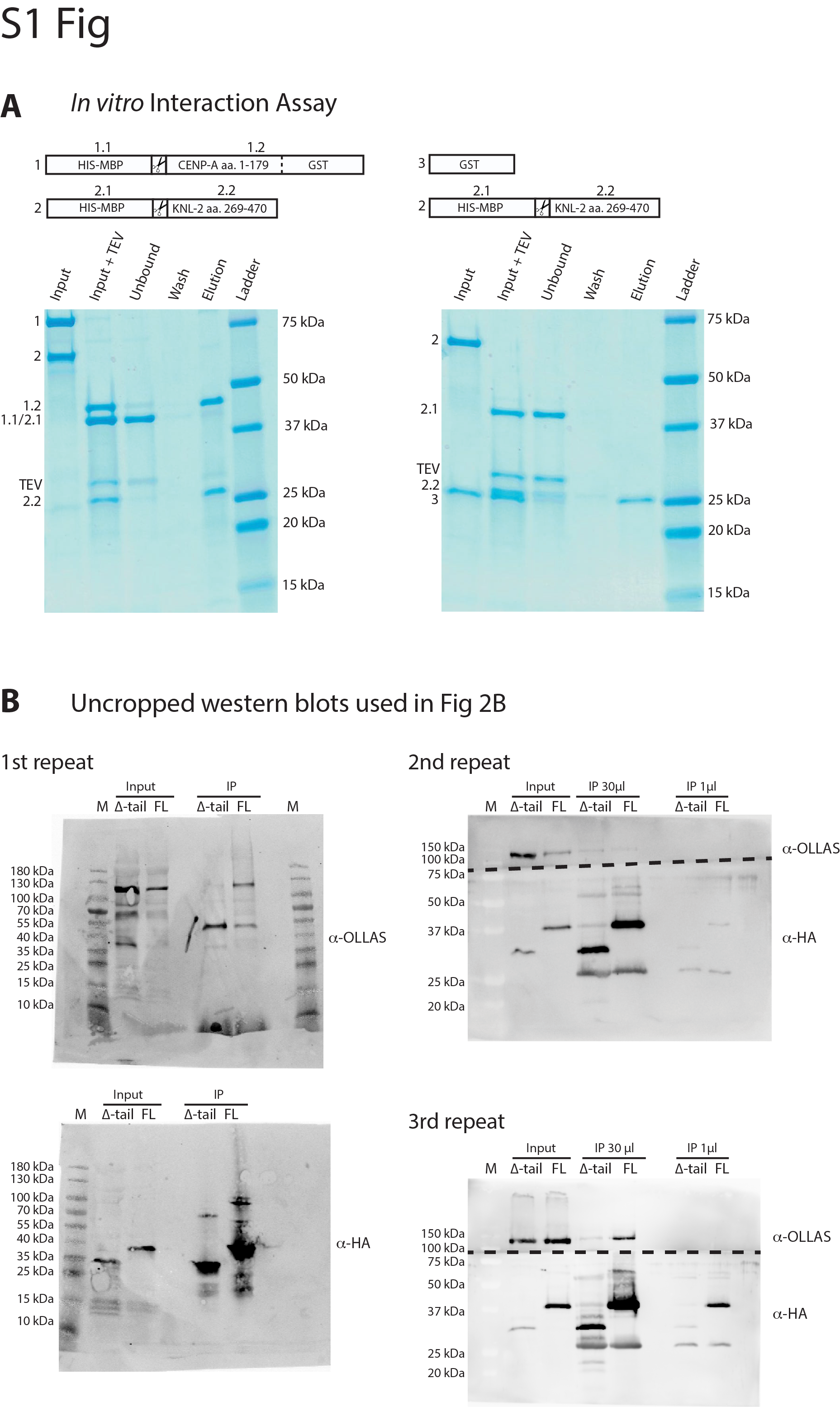

Supplement: S1 Fig — (A) In vitro interaction of CENP-A N-terminal tail and KNL-2 central domain. CENP-A N-terminal tail (aa 1–179) fused to GST, GST alone, and the KNL-2 central region (aa 269–470) were purified from bacteria. To aid solubility, a HIS-MBP tag was fused to the KNL-2 and CENP-A peptides. The MBP tag was removed by TEV cleavage (scissors in cartoons) before the pull-down using GST beads. The GST::CENP-A N-terminal tail (left) co-precipitates the KNL-2 central fragment, while GST alone (right) does not. (B) Uncropped western blots used to generate Fig 2B. For the first repeat, samples were loaded in duplicate for detection with anti-OLLAS and anti-HA antibodies. For the second and third repeats, western blot membranes were cut at a cutoff of about 80 kDa (dashed lines), and the parts were incubated with anti-OLLAS and anti-HA antibodies, respectively. CENP-A, centromere protein A; FL, full-length; IP, immunoprecipitation; TEV, tobacco etch virus. (TIF) [file pbio.3000968.s001.tif]

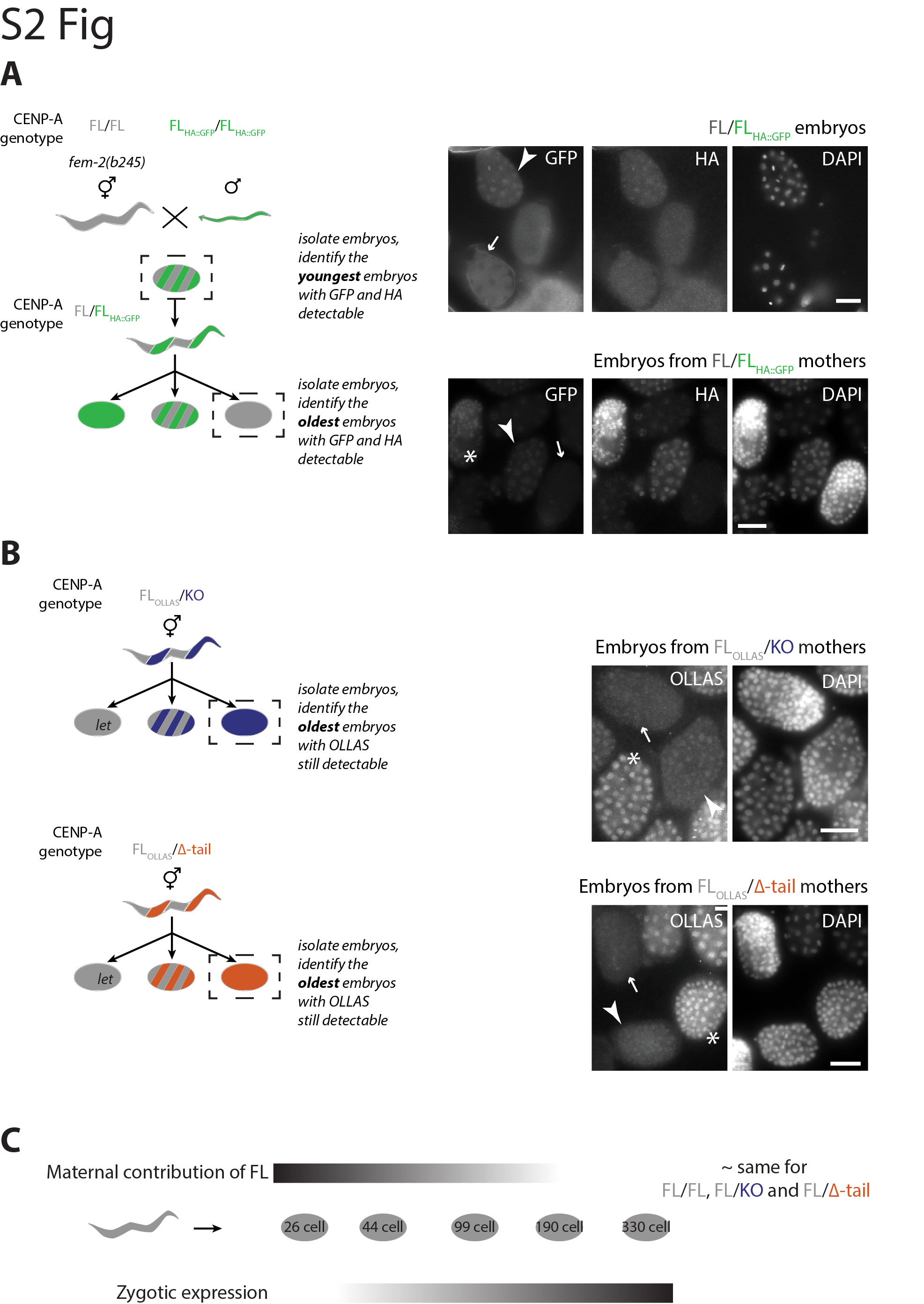

Supplement: S2 Fig — Cartoons show the different CENP-A alleles (GFP-tagged FL, green; FL, gray; KO, dark blue; Δ-tail, orange) in heterozygotes (striped) or homozygotes (full). Dashed boxes highlight the embryos relevant for the analysis. In the fluorescence images, arrowheads point to embryos with visible CENP-A FL protein, arrows point to embryos without visible CENP-A FL protein, and asterisks mark embryos with zygotically expressed CENP-A FL protein that obscures the analysis of maternal contribution. (A) Crossing males homozygous for GFP- and HA-tagged CENP-A FL to feminized fem-2 hermaphrodites homozygous for untagged CENP-A FL results in heterozygous F1 progeny that allow detection of the onset of zygotic expression of CENP-A (top). Self-fertilization of these F1 heterozygotes results in 25% embryos homozygous for untagged CENP-A FL that allow determination of the limits of maternal CENP-A FL contribution (bottom). (B) Determination of maternal CENP-A FL contribution as in (A), but CENP-A FL is OLLAS-tagged instead of GFP- and HA- tagged and is analyzed in the context of CENP-A KO or CENP-A Δ-tail instead of untagged CENP-A FL. Self-fertilization of heterozygotes results in 25% embryos homozygous for untagged CENP-A FL that allow determination of the limits of maternal CENP-A FL contribution. Scale bars represent 20 μm in (A) and (B). (C) Summary of the analysis described in (A, B), showing that maternally contributed CENP-A protein is detectable until the 100–200 cell stage, and zygotic expression is first detected at the 30–40 cell stage. Embryonic stages from beginning (26 cells) to end (330 cells) of gastrulation reflect those shown in the Wormatlas (www.wormatlas.org). CENP-A, centromere protein A; FL, full-length; KO, knockout. (TIF) [file pbio.3000968.s002.tif]

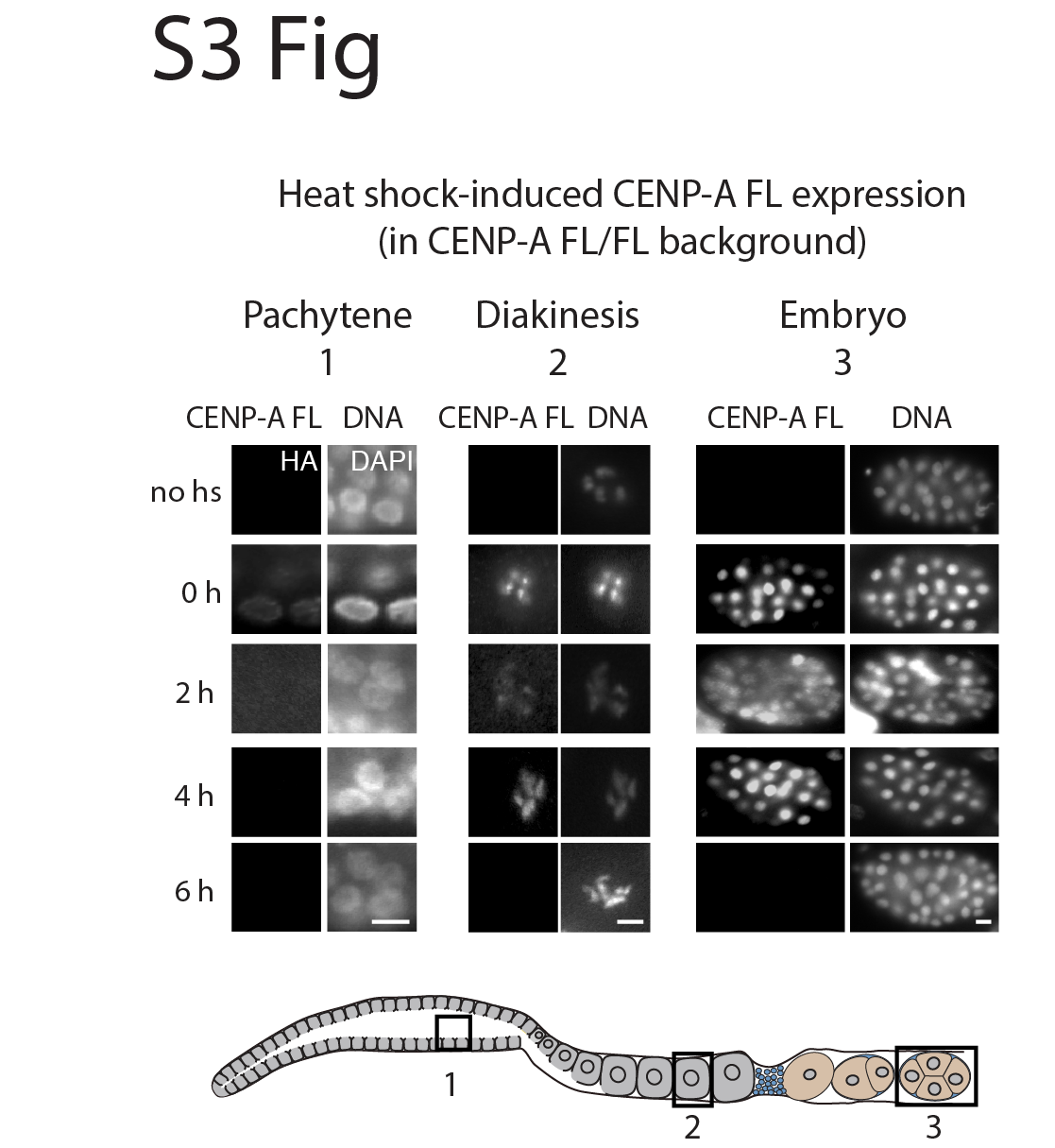

Supplement: S3 Fig — Pachytene and diakinesis nuclei of adults and embryos (highlighted in the cartoon image of the gonad) before and at the indicated time points after heat shock are shown. The images are shown for heat shock−induced HA-tagged CENP-A FL protein in a non-tagged CENP-A FL/FL background, because the CENP-A Δ-tail protein in the strain used for Fig 4D is also HA-tagged. Scale bars correspond to 5 μm. CENP-A, centromere protein A; FL, full-length; IF, immunofluorescence. (TIF) [file pbio.3000968.s003.tif]

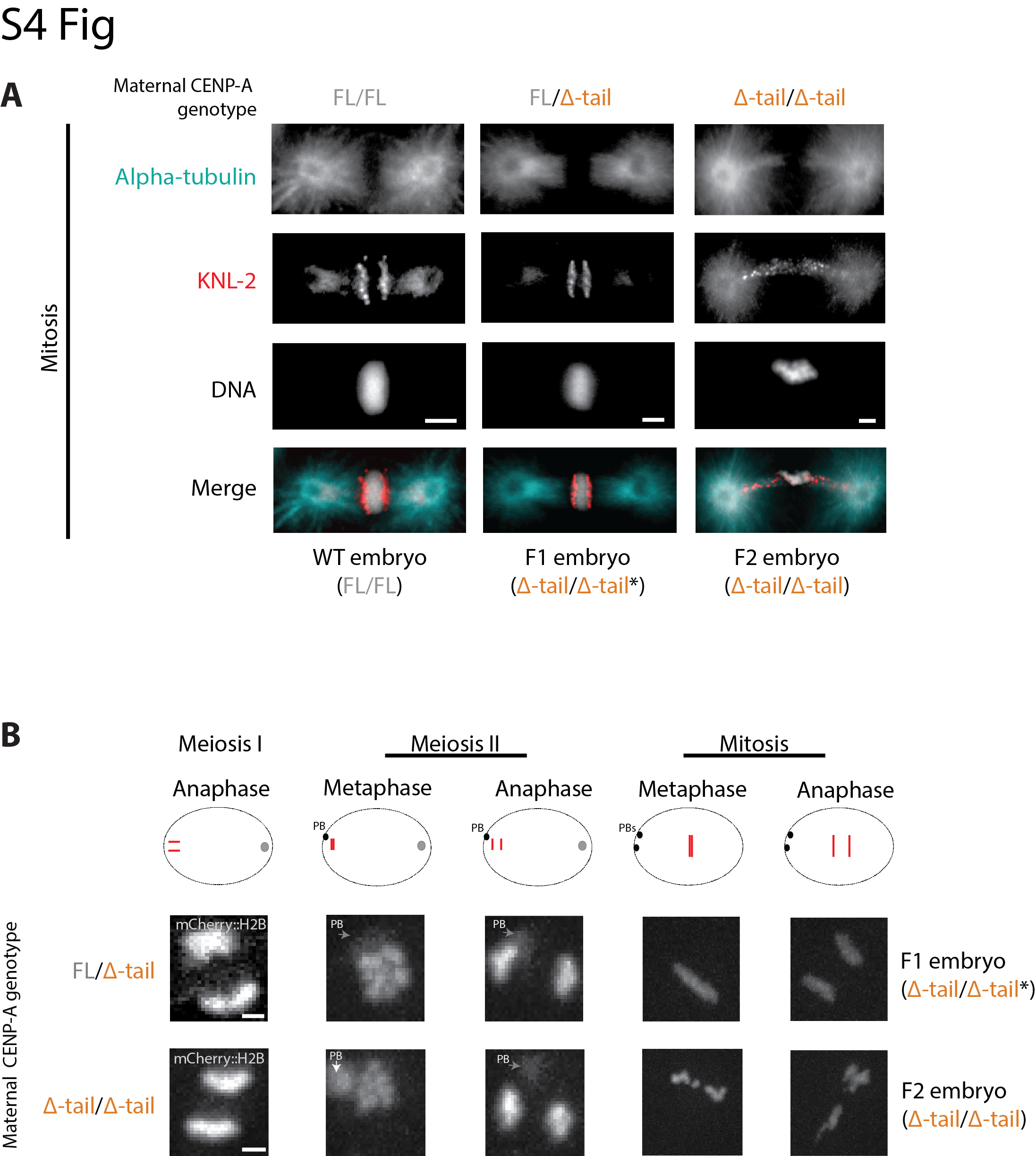

Supplement: S4 Fig — (A) Altered KNL-2 dynamics in mitosis of CENP-A Δ-tail homozygous F2 embryos. IF images depicting KNL-2 and α-tubulin at metaphase of early embryonic cell divisions in embryos derived from CENP-A FL/FL, FL/Δ-tail, or Δ-tail/Δ-tail maternal germ lines. (B) Meiosis is unaffected in CEN-P-A Δ-tail homozygous F2 embryos. Top, cartoon images of embryos with the chromosome stages corresponding to the live cell images in red. Bottom, still images of live cell recordings of embryos derived from CENP-A FL/Δ-tail or Δ-tail/Δ-tail maternal germ lines. H2B::mCherry was used to visualize chromosome segregation in meiosis I and meiosis II and mitosis of the first embryonic cell division. Polar bodies are labeled with PB and arrows. CENP-A FL/Δ-tail and Δ-tail/Δ-tail F1 offspring are indistinguishable as early embryos; therefore, the F1 genotype is marked with an asterisk. Scale bars represent 2 μm. CENP-A, centromere protein A; FL, full-length; IF, immunofluorescence; WT, wild-type. (TIF) [file pbio.3000968.s004.tif]

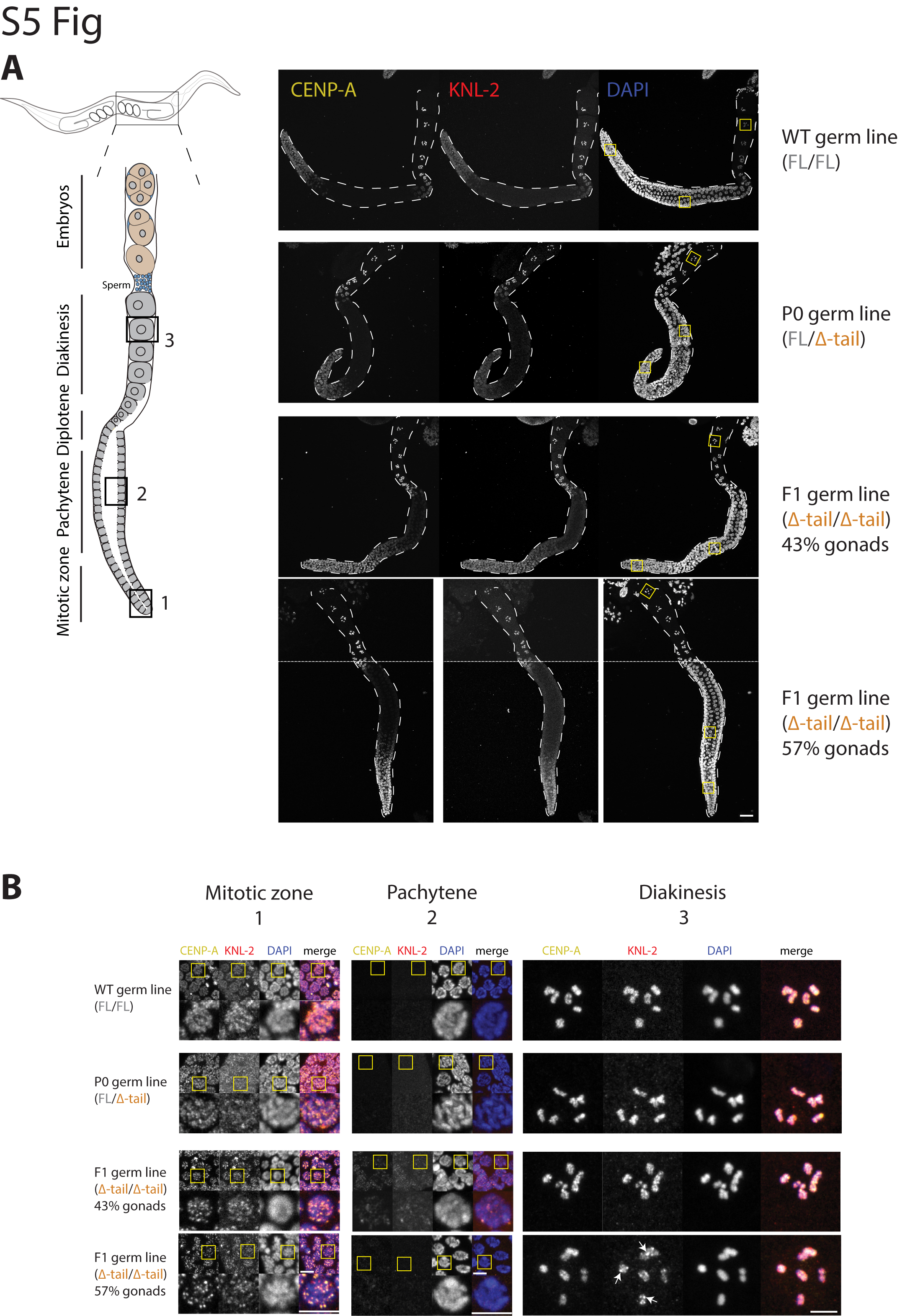

Supplement: S5 Fig — IF images showing the patterns of KNL-2 and CENP-A FL and Δ-tail, counterstained with DAPI, in adult germ lines of CENP-A FL/FL (WT control), FL/Δ-tail (P0), and Δ-tail/Δ-tail (F1) animals. (A) Whole gonads dissected from adults, and cartoon describing the different regions of the gonads. The extension of distal CENP-A Δ-tail and KNL-2 signal into the pachytene region in CENP-A Δ-tail homozygotes is visible in 43% of the gonads analyzed. Yellow boxes highlight the regions shown in (B). The dashed lines outline the gonads, and the dotted lines indicate where 2 images of the same gonad have been merged. (B) Zoomed images of nuclei in the mitotic zone, the pachytene region, and diakinesis oocytes, as highlighted in the cartoon image of the germ line in (A). Yellow boxes highlight individual nuclei that are enlarged below. Arrows indicate the KNL-2 foci in diakinesis. Scale bars represent 20 μm in (A) and 5 μm in (B). CENP-A, centromere protein A; FL, full-length; IF, immunofluorescence; WT, wild-type. (TIF) [file pbio.3000968.s005.tif]

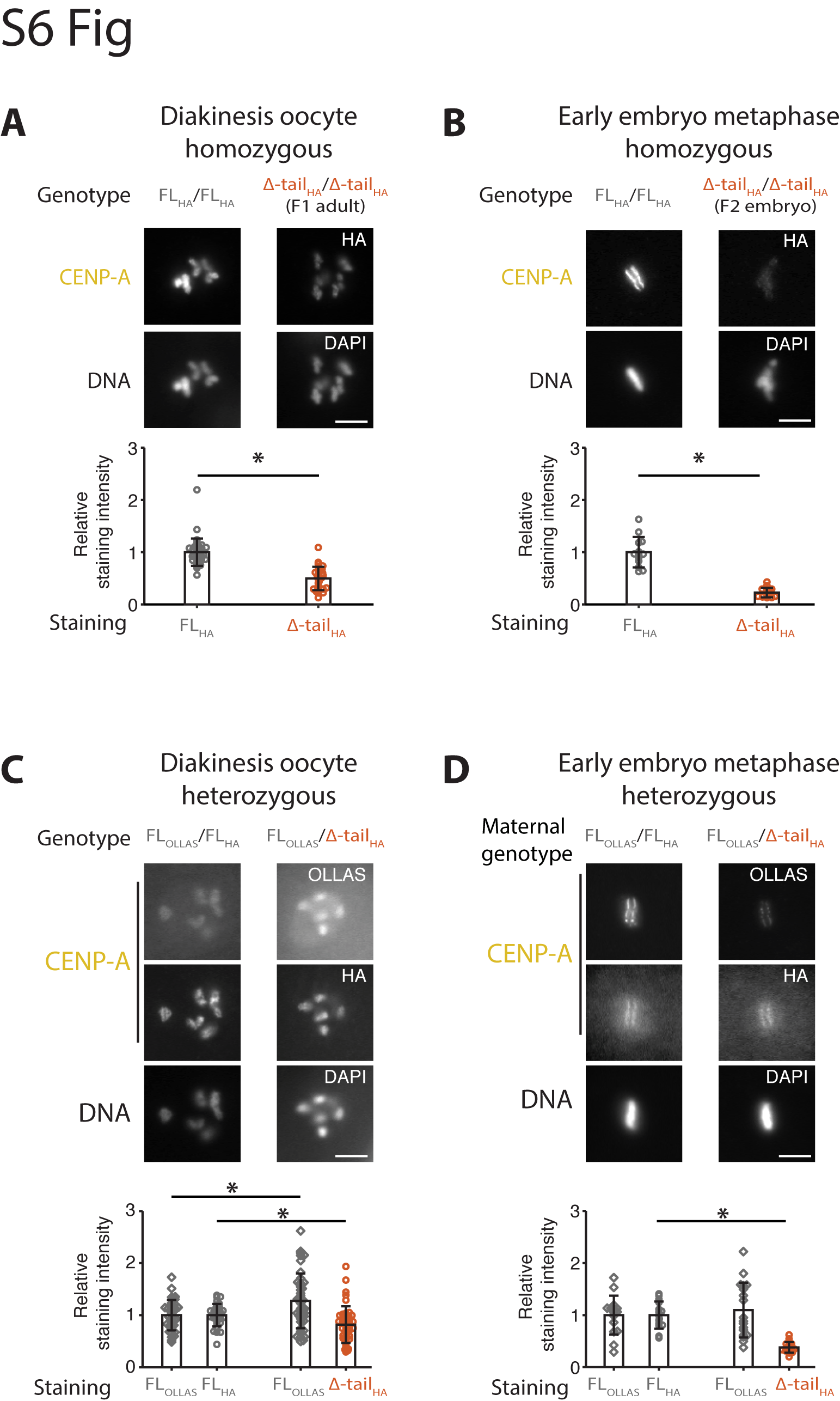

Supplement: S6 Fig — CENP-A levels were compared in CENP-A FL or Δ-tail homozygous (A, B) and in CENP-A FL/Δ-tail heterozygous (C, D) animals. In homozygous animals, CENP-A FL or CENP-A Δ-tail is HA-tagged, whereas in heterozygous animals, one copy of CENP-A FL is OLLAS-tagged, and the other copy is either HA-tagged CENP-A FL or HA-tagged CENP-A Δ-tail. CENP-A levels were determined and quantified in diakinesis nuclei (A, C) and on metaphase plates in early embryos (B, D). N = 27–43 oocytes and 13–18 embryonic metaphase plates from 3 independent IF experiments. In each IF experiment, the mean level of CENP-A FL in CENP-A FL homozygotes was set to 1. Error bars show the standard deviation of the mean. Asterisks denote statistical significance, determined by using a Student t test. Scale bars represent 5 μm. The data underlying all the graphs can be found in S1 Data. CENP-A, centromere protein A; FL, full-length; IF, immunofluorescence. (TIF) [file pbio.3000968.s006.tif]

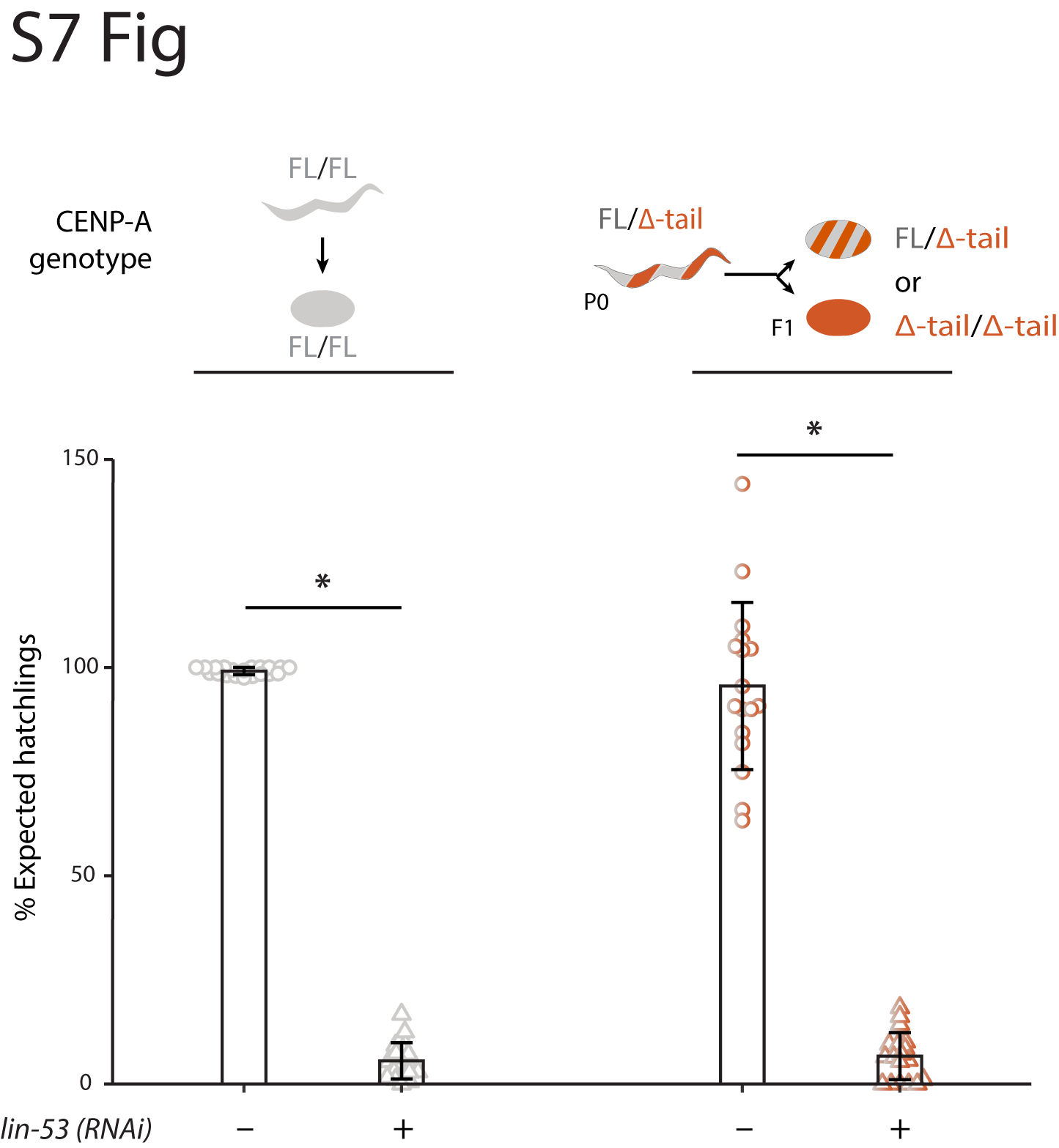

Supplement: S7 Fig — Cartoons show the different CENP-A alleles (FL, gray; Δ-tail, orange) in heterozygotes (striped) or homozygotes (full). Percentage of expected hatchlings in control animals and upon LIN-53 depletion in CENP-A FL homozygous and CENP-A FL/Δ-tail heterozygous mothers are shown. RNAi-mediated depletion of LIN-53 was induced by feeding in parental animals from L4 larval instar. CENP-A FL/Δ-tail heterozygotes contain the balancer allele, and CENP-A FL homozygous embryos are therefore inviable. N = 17–20 broods from 3 independent RNAi experiments. Asterisks denote statistical significance, determined by using a Student t test. The data underlying all the graphs can be found in S1 Data. CENP-A, centromere protein A; FL, full-length; RNAi, RNA interference. (TIF) [file pbio.3000968.s007.tif]
