# Supplementary material for: Transgenerational inheritance of centromere identity requires the CENP-A N-terminal tail in the C. elegans maternal germ line
Source: PLoS Biol. 2021 Jul 6;19(7):e3000968. doi: 10.1371/journal.pbio.3000968 (PMC8259991; doi:10.1371/journal.pbio.3000968)
Supplement: Attachment — Submitted filename: Response_to_Reviewers.docx [file pbio.3000968.s018.docx]

**Point by point response to the Reviewers**

**Editor**

You will see that all three reviewers agree that the finding that CENP-A has different roles in the germ line and embryo is interesting. Nevertheless, both Reviewers 2 and 3 raise several concerns regarding the clarity of the text and the fact that some of the conclusions are not fully supported by the data. They suggest experiments aimed to address these issues that you should perform.

We thank the reviewers and the editor for their positive assessment of our work, and for the chance to submit a revised version of our manuscript. For the current version, we have revised the text to improve clarity, revised the figures (especially Fig 3, 6, 7, S5) and added additional data (Fig 4, S2, S6, S7) that support our conclusions. The main additions include:

- Determination of the limits of maternal CENP-A contribution (mid-gastrulation) and the start of zygotic expression (early gastrulation), shown in new S2 Fig.
- Quantification of CENP-A FL and ∆-tail levels in homozygous and heterozygous cells, shown in new S6 Fig.
- Genetic demonstration that post-gastrulation, CENP-A ∆-tail is as functional as CENP-A FL in mitosis, shown in new Fig 4B.
- Demonstration that the lethality in CENP-A ∆-tail homozygous F2 embryos can be rescued by a pulse of CENP-A FL expression, with a time delay in the observed rescue that suggests that the protein is required in the maternal germ line (new Fig 4D, S3 Fig).
- Extension of the observation that chromatin association of CENP-A ∆-tail depends on LIN-53, shown in revised Fig 6 and new S7 Fig.

**Rev. 1:**

This paper investigates the very important issue of how centromeres are assembled and maintained in the germline and how they are inherited from one generation to the next. Specifically, this paper investigates the CENP-A inheritance in worms. Worms display unusual properties with respect to CENP-A inheritance. Firstly, sperm (paternal) chromosomes are devoid of CENP-A and it is reloaded only after fertilisation. Secondly, CENP-A is removed at the mitosis-to-meiosis transition the germline and it is reloaded in diplotene of meiosis. Therefore, it is apparent that different from 'template' models of inheritance that have been proposed in other organisms, centromeres must be established de novo at both of these stages in worms. Here the authors convincingly show a key role for the CENP-A N terminus, as well as KNL2 and LIN-53, in de novo centromere assembly in the germ line.

I have very little criticism of this paper. It is a very interesting read and presents some unexpected results in a clear and concise manner. All experiments are well designed, well executed and results are well interpreted. The results have far reaching implications with respect to mechanisms of epigenetic inheritance in the germline.

We thank the reviewer for the kind feedback and the constructive comments.

I have only three very minor queries/comments:

1. In Figure 2, the authors convincing show that the CENP-A N terminus is both necessary and sufficient for interaction with KNL2 using both in vitro and in vivo approaches. In Figure 2B, the error bar for KNL2 enrichment in the CENP-Aaa1-99 pulldown is quite large and only two replicates were performed. The authors should clarify how this enrichment was calculated. In Figure 2C, lanes 4 and 5 appear to be switched. Shouldn't FL-CENP-A run higher than the truncated version?

We have added additional western blots replicates for Fig 2B (used to be 2C), and clarified how the enrichment was quantified in the figure legend (lines 1052-1055). The enrichment is determined as the IP ratio of KNL-2/CENP-A divided by the input ratio of KNL-2/CENP-A, with this value set to 1 for CENP-A FL in each experiment. We don’t think that the absolute values are meaningful, but the comparisons are. Repetition of the blots for the original Figure 2B (KNL-2 interaction with H3.3 chimeras) ended up increasing the variability of the results. We believe that this variability is the result of technical problems, but we were unable to solve these and to robustly quantify the interaction. We therefore decided to remove this panel from the figure, also in response to Reviewer 3 comment 11. We note that the interaction of KNL-2 with the CENP-A N-terminal tail has recently also been described in a preprint from the Deasi lab (<https://www.biorxiv.org/content/10.1101/2020.12.28.424576v1>), which we now cite (citation 34).

The annotations in Fig 2B (used to be 2C) were indeed swapped, we apologize for the mistake and have corrected it.

2. In Figure 4B, the authors perform live imaging of the first embryonic division in WT, F1 and F2 embryos. Can the authors comment on the observed defects in chromosome condensation which are visible in the F2 embryos and perhaps also in the F1 embryos? Is this phenotype expected to result from a lack of centromere specification?

F2 embryos indeed show condensation defects, as would be expected from centromere defects. Loss of CENP-A or KNL-2 has previously been shown to result in condensation defects (Maddox et al 2007, PMID: 17339379). This is visible in Fig 5A (used to be 4A), and we now mention it in lines 324-326. Fig 5B (used to be 4B) shows mislocalized kinetochore proteins by live cell imaging, and without a chromatin marker, condensation defects are difficult to assess in these experiments.

3. In Figure S4, the authors investigate CENP-A and KNL2 localisation in the delta tail/tail F1 germline. Both proteins display a punctate pattern in the mitotic zone and in diakinesis. Can the authors comment on whether it is the intensity of these foci that has increased or whether a relocalisation event has occur? Increased intensity might indicate enhanced assembly or stability of CENP-A/KNL2 at this time, which could be important for understanding the mechanism of de novo centromere establishment.

We assume the Reviewer is referring to Figure S5. We believe that these foci are the result of relocalization, possibly as a consequence of the loss of interaction between CENP-A ∆-tail and KNL-2. However, quantification of this redistribution is technically challenging. We now show full gonad pictures (S5A Fig) with zooms to show CENP-A and KNL-2 localisation in different germ cell stages (S5B Fig), also in response to Reviewer 2 comment 2 and Reviewer 3 comment 6. We addressed the question of CENP-A ∆-tail levels on chromatin (S6 Fig), and found that they were reduced compared to CENP-A FL, which could indeed indicate lower accessibility or stability (lines 397-405). This experiment was included also in response to Reviewer 2 comment 4. Despite the punctate patterns and lower CENP-A ∆-tail levels, germline proliferation of CENP-A ∆tail F1 homozygotes is normal, and we therefore think that the mislocalization is not the underlying cause for the segregation defects observed in the F2 embryos. We comment on this in lines 366-369.

**Rev. 2:**

Prosée et al report two main findings: 1. That a deletion of the N-terminal tail of the centromeric histone CENP-A is a maternal effect lethal allele and 2. That the defect that causes this embryonic lethality is a failure in specifically KNL-2 mediated de novo centromere establishment in the maternal germline. The role of the N-terminal tail in germline transmission has been reported in plants, where an N-terminal CENP-A tailswap with the conventional H3 tail results in uniparental elimination of the genome with the mutant CENP-A (Ravi and Chan, 2010). The difference is that in this case, the tail mutant of CENP-A appears to have a phenotype in the following F2 generation, typical of a maternal effect allele. This is an interesting observation, but the interpretation of the data remains unclear in some areas. The crucial data that demonstrates the requirement of re-establishment of centromeres in the maternal germline for F2 viability is in the supplemental section which gives me pause since that is critical for the main conclusions (indeed, needed to support the claims of the title of the paper!). In addition, the authors find that LIN53 (previously described as a general histone chaperone that also assists in CENP-A nucleosome assembly, Ref 31) is also involved in CENP-A chromatin association but this is not well developed in the paper. As it stands the paper has an interesting observation but to highlight its importance, the paper would require substantial restructuring, more compelling data interpretation, and additional clarification of aspects of the experimentation (one example are some of the key images in Figure 4B; see point #5, below). Specific comments to elaborate on this overall assessment are as follows:

We thank the reviewer for the kind feedback and the constructive comments.

Major comments:

1. The major claim of the paper is that the N-terminal tail of CENP-A is dispensable for mitosis during development but required for re-establishment in the maternal germline. However, in the absence of an initial template supplied by wild type maternal CENP-A (FL) templated chromatin, the N-terminus is also unable to support mitosis (since it apparently doesn't know where to assemble), so the term dispensable is confusing here (Fig 3A, C).

We agree with the reviewer that the language we used is not precise. We changed it to “but then its presence becomes dispensable for the ensuing centromere maintenance during development“ or “the presence of the CENP-A N-terminal tail is dispensable during development” (e.g. lines 34-35, 105-106, 165, 233-234, 1057).

Further, the somatic auxin depletion (3-day treatment in adults, S2B) does not affect hatch rates and could also be explained as the already established epigenetic identity in the P0 germline being maintained.

This is exactly our interpretation. We have moved the developmental AID experiment into main Fig 3C to juxtapose it with the germline AID experiment (following your recommendation below), and have revised the text describing it (lines 220-232). The main point of the somatic/developmental AID experiment is to show that mitosis during development is fully supported by CENP-A ∆-tail, and not by trace amounts of FL that are below detection level, but might persist through the development. This is now also supported by additional genetic crosses shown in new Fig 4B, showing that the embryonic lethality of CENP-A KO embryos can be rescued by zygotic expression of either FL or ∆-tail.

It would seem quite possible that the auxin depletion is not complete and the remaining CENP-A, however little, is sufficient to direct the mutant to the right genomic locations. A western to quantify the auxin depletion in the germline vs somatic depletion would help clarify this.

We realize that we did not explain the AID experiments sufficiently, and that there is confusion between the somatic and germ line AID experiments. A similar concern was raised in Reviewer 3 comment 2. We therefore now show the two experiments side by side in Fig 3C and D.

CENP-A FL is already undetectable in the adult F1 germ line by IF (Fig 3B) before the somatic/developmental AID experiment, and we doubt that western blots would be a more sensitive method of detection. The point of the somatic/developmental AID experiment is precisely to test if there are undetectable levels of CENP-A FL that may be sufficient to support mitosis until adulthood, and the results show that this is not the case. However, the depletion is sufficient to cause lethality/arrest of the control CENP-A FL homozygotes.

In the germline AID experiments, we indeed show that upon depletion, there is no CENP-A FL protein to define the right genomic locations, and therefore the F1 embryos are inviable (Figure 3D), indicating that the remaining residual amounts of CENP-A FL are not sufficient to direct the centromeres to the correct locations. We show that the depletion is almost complete by IF (Figure 3D), and western blots are unlikely to add additional information, and would technically be much more challenging.

Figure 3A, C and S2 are the most impactful observations in the paper in my opinion and should be further highlighted in the text. It would also be stronger, in my opinion to present them together in one main figure. The distinction between the germline re-establishment vs. maintenance made by the somatic vs germline depletion experiments and the F1 sterile hermaphrodites should ideally be more clear in a revised manuscript.

We agree with the reviewer, and we have now moved the panels of S2 Fig into the main figures, Fig 3C (used to be S2B) and Fig 4A and C (used to be S2A), and have revised the text accordingly.

2. All the experiments point towards a defect in de novo establishment of maternal centromere identity in the Tail mutant F1 germline. This is the crux of the paper (in the title) and the new advance that makes this paper different than the entire bulk of the plant studies on the N-tail. Therefore, it is quite surprising that the data that specifically demonstrates this is a supplemental figure (S5)! I strongly suggest moving that data into the main paper. Just looking at Fig S5 I do not see a clear difference between the P0 and F1 germline CENP-A other than the punctate nature of KNL-2. Since this is unclear, it would seem to be crucial that the authors provide images of the full germline (as in Figure 1) for all the genotypes in S5, specific nuclei in mitotic and diakinesis zones can be zoomed in on in insets. This will also help for the reader to appreciate the claim in line 290-292 that the CENP-A mutant removal is delayed.

We realize that our description of the results shown in S5 Fig was confusing. We now provide full gonad pictures (S5A Fig) with zooms (S5B Fig) to show CENP-A and KNL-2 localisation in different cell stages in the germ line, as suggested, also in response to Reviewer 1 comment 3 and Reviewer 3 comment 6.

However, we believe that the two main observations in this figure - the dotted appearance of CENP-A ∆-tail and KNL-2 in the distal F1 germ line, and the extension of ∆-tail into the pachytene region - are not underlying the centromere re-establishment defects that the paper mainly focuses on. The F1 CENP-A ∆tail homozygotes show normal development and a normal germline proliferation. Also, the pachytene staining of the CENP-A ∆-tail protein is not fully penetrant, as is now shown in S5 Fig, while the embryonic lethality of the F2 embryos is. We have revised the text (lines 356-375), but prefer to leave this figure in the supplement.

The authors have convincing data that this chromatin associated CENP-A is clearly not functioning in centromere identity since the outcome is drastic mis-segregation later in the embryo. But it is very confusing as to why the next section in the text after referring to S5 assumes that KNL-2 is required for de novo assembly in the germline when the diakinetic nuclei clearly have chromatin associated CENP-A. The authors explain that the KNL-2 mediated CENP-A assembly is the functional population which is clear from the lethality in the F2 generation but I am not sure why LIN53 mediated chromatin association is not regarded as assembly but is nonetheless designated as "maintenance". Since CENP-A is removed early in the germline, any chromatin association in the proximal germline has to be de novo assembly and not maintenance (see point 3, below).

We agree with the reviewer that the language was not precise enough. We have made it clear now that KNL-2 is required for CENP-A **FL** chromatin association (lines 380-390), as shown in our KNL-2 germline depletion experiment in Fig 6A. However, CENP-A ∆-tail, which no longer interacts with KNL-2, still associates with chromatin, even in the absence of KNL-2 (Fig 6B, lines 391-396). We showed that this chromatin association of CENP-A ∆-tail depends on LIN-53 in embryos, and we now extend this finding to diakinesis oocytes (Fig 6C, lines 413-427).

We hypothesize that the N-terminal tail and its interaction with KNL-2 is required for the deposition of CENP-A at the correct genomic regions (centromere), while the chromatin association of both FL and ∆-tail depends on LIN-53. Without the N-terminal tail, we think that the chromatin loading becomes random (non-centromeric), leading to the aberrant localization pattern in F2 embryos as shown in Fig 5A (used to be Fig 4A). Since we show that the ∆-tail protein is sufficient for centromere function during development, and its presence on chromatin requires LIN-53, we have used the term “maintenance”. But we agree with the reviewer that we cannot exclude that LIN-53 is also involved in *de novo* establishment. In fact, this is likely the case, and LIN-53 is also required for chromatin association of CENP-A FL, and likely involved in the establishment of the faulty centromere identity in the F1 proximal germ line.We now include the possibility that LIN-53 is also involved in establishment in the results section (lines 423-425), and explain our hypothesis in the discussion (lines 514-539), also in response to reviewer 3 comment 7.

3. The title of Figure 5 "Chromatin association of CENP-A depends on LIN53 and not KNL-2' is confusing. What do the authors mean by chromatin association? All nucleosomes are chromatin bound including the KNL-2 population apparent from the fact that the P0 and F1 germlines do not look different in CENP-A localization unless the levels are lower. I think what the authors mean is that the KNL-2 assembled population is the only functional population at least for de novo establishment of centromere identity. The result in this section brings up the interesting idea that even in holocentrics the location of CENP-A matters. The KNL-2 mediated de novo assembly (Figure 5) must be directed to certain locations perhaps more conducive for establishing epigenetic memory whereas the LIN53 mediated assembly could be more random; this should be discussed in the text.

We agree with the reviewer that the language was not precise enough. We have changed the title of Fig 6 (used to be Fig 5) to “CENP-A ∆-tail is maintained on chromatin by LIN-53 in absence of the interaction with KNL-2”. We show that while chromatin association of CENP-A FL depends on KNL-2, this is not the case for CENP-A ∆-tail (Fig 6A, B). We also show that chromatin association of both CENP-A FL and ∆-tail depend on LIN-53 (Fig 6C). We indeed think that only the KNL-2-assembled population is capable of setting centromere identity, and that indeed CENP-A ∆-tail will be mislocalized by LIN-53-dependent assembly, and we have added these points to the discussion (lines 514-539). It is worth stressing that this difference holds true only for re-establishment of centromeres, not maintenance, since the ∆-tail protein alone is able to maintain fully functional centromeres for one generation in the F1 animals.

Does germline specific knockdown of lin53 yield viable embryos? Do the F1 embyros obtained by treating FL/Tail P0 hermaphrodites with lin53 RNAi develop?

Lethality of LIN-53 depletion has been previously demonstrated (Lee et al 2016, PMID: 26904949**).** We have added LIN-53 RNAi depletion experiments and showed that these result in reduced embryonic viability for both CENP-A FL/FL and FL/∆-tail maternal genotypes, at comparable levels (new S7 Fig; lines 425-427).

4. Quantifying the levels of CENP-A, like with the embryos in Fig 5 A and B, to better understand the two pathways in the germline where the action happens would help support the conclusions made in the paper. Quantification of the levels of FL vs tail in the P0, F1 and F2 embryos is also going to be informative. Is the Tail just generally less efficient in nucleosome assembly?

We quantified CENP-A FL and ∆-tail levels in diakinesis nuclei and early embryos using IF images, for both homozygous and heterozygous cells, and the results are reported in new S6 Fig and in lines 397-405. We found that CENP-A ∆-tail levels are significantly lower compared to FL, and that this reduction was more pronounced in homozygous cells compared to heterozygous cells. These results indicate that nucleosomes containing the CENP-A ∆-tail are either less efficiently assembled or less stable. Nevertheless, the levels of CENP-A ∆-tail are sufficient to support mitosis during development and germ line proliferation in F1 homozygotes.

5. In figure 4B, unless I am confused by what is shown, there appears to be an image repetition in the metaphase panel for the homozygous F2 tail mutant embryos. The GFP::KNL-2 and the GFP::ROD1 panels look nearly identical for those mutant embryos.

We apologize for this mistake. The ROD-1 movie still was indeed inserted instead of the KNL-2 movie still in the ∆tail/∆tail panel. We have replaced the KNL-2 metaphase still, thereby also resolving the discrepancy between Fig 5A and B (used to be Fig 4A, B) pointed out in Reviewer 3 comment 13.

6. Shouldn't the model include LIN53 mediated maintenance since it appears to be important for the downstream function of the centromeres once KNL-2 mediated de novo assembly is established in the germline? In addition, although Figure 6B is explained well in the text, the figure does not demonstrate the germline epigenetic inheritance posited by the authors. The authors provide two scenarios, function vs presence of protein but a conclusion consistent with their experiments (and those of many others) is that epigenetic inheritance is essentially the presence of protein, just assembled correctly in the genome versus existing as a maternal pool. I think the authors would like to make the distinction between maternal contribution (classical maternal effect genes) versus maternal epigenetic inheritance, which refers to all chromatin dependent epigenetic marks inherited through the parent. This should be clarified, as the defect the authors want to highlight is in the maternal

germline epigenetics and not a consequence of defective maternal protein pool in the embryo.

We have revised the model in Fig 7A to show that LIN-53 likely plays a role in both centromere establishment and maintenance. In addition, we agree with the reviewer that we wanted to make the distinction of maternal contribution vs maternal epigenetic inheritance, and hope that this is now more clear from the revised Fig 7B.

7. The inclusion of the recombinant protein data (Fig. S1) is questionable. If it was robust and well controlled it would be in the main figure set. As is, it seems unreliable and so it is relegated to the supplement. This doesn't seem like a strategy that engenders a lot of faith in the conclusions of the paper more broadly.

We show the recombinant protein data in the supplement to avoid overloading the main figure, and not because we have concerns about the result.

Minor comments:

8. N should be listed throughout. It is unclear what the dots represent in many places. The data are all provided as percentages, so the actual number of worms must be added to all relevant figures including the number of repeats (or P0 parents used). In addition, for the somatic depletion of FL protein, when was the counting performed, at the end of 3 days?

We have added N numbers and definitions of the individual data points to the figure legends. For the somatic AID experiments, embryos were placed on auxin plates and counted, and worms were indeed scored after 3 days of auxin-mediated CENP-A FL depletion during development. We have clarified this in lines 224-225 and 697-701.

9. It would also help broad non-germline readers if the schematic for germline development is put in the context of when maternal contribution ends in the worm. This would make it clear that there is no maternal FL protein available in the F1 homozygote germline. This point is mentioned in Line 195-197 but citing Figure 3B-F1 diakinetic nucleus would also help.

A similar point was raised in Reviewer 3 comment 5. We have analyzed the range of maternal contribution and zygotic expression and found that maternal CENP-A becomes undetectable in mid-gastrulation (100-200 cell embryo), while zygotically expressed CENP-A becomes first expressed at the beginning of gastrulation (30-40 cell embryo). These results are reported in new S2 Fig and described in lines 195-218.

10. Suggested re-organisation of the paper: Perhaps the authors could consider moving S5 to Figure 4 and Figure 4 to S5. Figure 4 currently enumerates how the embryos are dying by mis-segregation, which is important but expected for a CENP-A mutant. The main result is that the defect is upstream in the female germline and should be highlighted in the paper.

For reasons explained under comment 2, we kept S5 Fig in the supplement. To strengthen the conclusions that the defects originate in the germ line, we have added Fig 4, showing that CENP-A KO embryos can be rescued by zygotic expression of CENP-A FL or ∆-tail protein, while F2 CENP-A ∆-tail embryos cannot be rescued by zygotic expression of FL (new Fig 4B, C). In the same figure, we also show that the F2 embryos can be rescued by a pulse of FL protein in the mother, and this rescue requires a delay that likely corresponds to the time required for the proximal germ cells to become embryos (new Fig 4D,S3 Fig).

We agree that the phenotypes shown in Fig 5 (used to be 4) may be expected from a CENP-A mutant, but we think it is important to show that the same mutation can have different phenotypes in the F1 vs F2 homozygous embryos.

11. All nuclear fluorescence quantifications should be normalized to the control (WT and/or without auxin) so as to make it easier for the reader to gauge levels of decrease.

We followed the Reviewer’s suggestion, and all image quantifications are now normalized by setting the control of each experiment to 1 (Fig 2B; Fig 6; S6 Fig).

12. For table S1, it would be really helpful if the strains used in the different figures were annotated in the "Comments" column. Also, the germline and soma specific drivers used should be specified there for easy access for the reader.

We have listed the figures in which the strains have been used in S1 Table. We have also listed the original TIR1 driver strains with a more detailed description of the transgenes they contain.

13. In the discussion of the paper, it would be nice to discuss these results in the context of and contrasted to the plant N-terminal tail studies and highlight how this instance compares (elaborate lines 398-401). Also, the N-terminal tail of mammalian CENP-A has also been shown to be dispensable (if other parts of CENP-A are present) for maintenance (Ref 51) but indispensable for establishment at an ectopic site (Ref 52). It would seem to help the author's case about broad biological implications of their findings to make more mention of all of these prior findings.

We have extended the discussion of the N-terminal tail in other organisms (lines 491-512).

14. Line 277 sentence needs references and to remind again which systems CENP-A has been shown to be dispensable for meiosis.

We have changed the phrasing to “dispensable for chromosome segregation during meiosis in C. elegans” and added the missing reference (Monen et al 2005, PMID: 16273096, reference 28, line 348), also in response to Reviewer 3, comment 15.

15. Line 346: I do not believe Mis18 was tested in Ref 25.

We thank the Reviewer for pointing out this mistake. We have removed this subclause (line 448).

**Rev. 3:**

The paper set out to study how CENP-A is inherited trans-generationally. This paper presents work carried out in the nematode C. elegans to identify the N-terminal tail of CENP-A as a critical domain for the interaction with the conserved kinetochore protein KNL-2, and argued that this interaction plays an important role in setting the centromere identity in the germ line (diakinesis stage).

They first used yeast two-hybrid assay to investigate the interplay between CENP-A and KNL-2 and found that aa 1-122 of the CENP-A N-terminal tail are sufficient for the interaction with KNL-2. In vivo, CENP-A N-terminal tail (aa 1-99)-HIS-72 (a non-essential histone H3.3 homologue) chimeras was sufficient to mediate interaction with KNL-2 in co-IP experiments.

Then they constructed and maintained a CENP-A N-terminal tail truncation allele heterozygous worm strain in the context of a full-length copy of CENP-A balancer allele. They used this balancer strain (CENP-A FL/∆-tail) P0, the F1 and F2 embryos and germ line to investigate the function of the N-terminal tail, the interaction with KNL-2, and the localization interdependency with KNL-2.

They found that the F1 generation of the balancer strain homozygous for CENP-A ∆-tail were fully viable and developed indistinguishably from wild type worms and showed no obvious germline proliferation defects, while the F2 embryos laid by the homozygous F1 were all inviable due to severe chromosome segregation defects. So, they conclude that the N-terminal tail of CENP-A is dispensable during development (embryogenesis), but is required in germ line for de novo centromere establishment in each generation. However, the presence of FL CENP-A in ∆-tail homozygous F1 embryos (Fig 3B) makes it difficult to make the conclusion (see major comments).

By using the AID system, they selectively depleted germline full-length CENP-A in P0 and observed severe chromosome missegregation in all F1 embryos. Depleting the P0 somatic CENP-A deos not cause defect in ∆-tail homozygous F1. Crossing in a CENP-A full-length gene copy through the paternal germ line does not rescue F2 embryonic lethality. With these results they conclude that maternally provided full-length CENP-A is required for de novo centromere establishment in the maternal germ line.

In the Diakinesis oocyte (proximal germ line), FL CENP-A loading is dependent on KNL-2, but not vice versa, suggesting that KNL-2 acts upstream of CENP-A in the de novo establishment of centromeres. Interestingly, CENP-A ∆-tail remains on chromatin upon depletion of KNL-2 in F1 CENP-A ∆-tail homozygotes in a KNL-2-independent manner. However, it is still unclear how the N-terminal CENP-A and KNL-2 contribute in de novo centromere establishment in detail. In addition to immunofluorescence, if the authors can show how the CENP-A ∆-tail becomes mislocalized on the genome (or mislocalized with other centromeric proteins), it may give more clue to the mechanism.

Through the elegant genetic design of the balancer strain (CENP-A FL/∆-tail), AID strains and accompanied biochemistry and imaging analyses, this study has helped to elucidate the interaction of KNL-2 with full-length and truncated CENP-A in vivo, and also the function of this interaction in CENP-A localization in the C. elegans proximal germline and the subsequent mitosis. In summary, it is exciting to see this work trying to elucidate the different role of CENP-A, KNL-2 and their interaction in the germ line vs. in embryos. However, it is not entirely convincing. If the authors can tone down on their conclusion on the germline specificity and on the "functional inheritance" mechanism, and just focused on their solid interaction and dependency results, it is still an interesting study with great contribution to the field. The observed phenotypes provoke insights and deserve further mechanistic investigation. Further work will be required to elucidate why and how the

interaction between KNL-2 and the CENP-A N-terminal tail can establish de novo centromere in germline and how to transmit this centromere identity into the next generation. Some questions are listed below.

We thank the reviewer for the kind feedback and the constructive comments. We have addressed some of the concerns with additional experiments to strengthen conclusions about the distinct roles of CENP-A in the germ line and during development. We fully agree that it will be of great interest to understand how and why the truncated CENP-A is functional in mitosis in F1, but not F2 animals, and how the interaction with KNL-2 contributes to centromere establishment. However, addressing these questions will require extensive new lines of experimentation, and we therefore feel that they are beyond what we can show in this current manuscript.

Major:

1. For the co-IP experiment, the authors used embryo extract. However, if the authors are mainly interested in de novo centromere establishment in the diplotene stage, shouldn't they also isolate germline extract (or use sterile fem-1 mutants?) to perform the co-IP?

We agree that it would be interesting to repeat the co-IP experiments from germ cell extracts. Unfortunately we were not able to adapt the protocol for this during the revision. Working with whole worms reduces the yield (proportionally less cells expressing CENP-A and KNL-2), and increases the background (presumably because the gut contents are released into the samples). Dissecting out germ lines was not possible due to the large amounts of input material required. However, we are confident that CENP-A and KNL-2 also interact in the germ line, based on our genetic and AID depletion experiments. Additionally, we note that the interaction of KNL-2 with the CENP-A N-terminal tail has recently also been described in a preprint from the Deasi lab (<https://www.biorxiv.org/content/10.1101/2020.12.28.424576v1>), which we now cite (citation 34).

2. Page 9 line 205 to 213

I do not understand how the AID experiment can exclude the possibility that "undetectable amounts of full-length CENP-A that could support development" in the N-tail homozygous F1 that is inherited from P0. The AID experiment shows that if FL is depleted in P0 germline, Degron::FL/tail F1 cannot hatch (no detectable FL in heterozygous diakinetics). What is the signal in Fig 3D FL CENP-A?

We realize that we did not explain the AID experiments sufficiently, and that there is confusion between the somatic and germ line AID experiments. A similar concern was raised in Reviewer 2, comment 1. We therefore now show the two experiments side by side in Fig 3C and D.

In the germline AID experiment, CENP-A FL is depleted in adult P0, leading to inviable progeny. The CENP-A FL signal in the embryo in Fig 3D is likely the result of zygotic expression. We now mention this in the text (line 257)

In the developmental/somatic AID experiment, embryos are placed on auxin plates, and CENP-A FL is depleted during development. If undetectable CENP-A FL protein was responsible for supporting the development of the F1 CENP-A ∆-tail homozygotes, developmental AID should lead to their arrest, as it does for CENP-A FL homozygotes (Fig 3C). This is not the case, and we therefore conclude that CENP-A ∆-tail alone is able to support mitotic chromosome segregation in F1. We have revised the text describing the somatic (lines 220-232) and germline (lines 251-260) AID experiments. See also response to comment 3.

3. On the other hand, if FL is depleted only somatically, Degron::FL/tail F1 can hatch (Fig. S2B quantification, but no IF image). For somatic depletion, are P0 in adult stage and what promoter is used? It means there will be maternal load FL CENP-A in F1 embryos? So again, it is not too surprising that they survive.

See also response to comment 2. For the somatic/developmental AID experiments, F1 and control embryos were directly placed on auxin plates, P0 animals were not treated.

The point of the somatic/developmental AID experiment was to confirm that CENP-A ∆-tail is able to support mitosis during development and germline proliferation in absence of CENP-A FL protein, a result that we find surprising.

CENP-A FL is already undetectable without AID in late F1 CENP-A ∆-tail homozygous embryos (new S2B Fig) and adults (Fig 3B), but we wanted to exclude the possibility that trace amounts of CENP-A FL persist and confound the interpretation of the F1 survival. We therefore depleted CENP-A FL during development and showed that while this is detrimental to CENP-A FL homozygotes, it does not affect CENP-A ∆-tail homozygotes. Because CENP-A FL is already below detection limit in F1 adults (Fig 3B), we did not attempt to detect it by IF after AID-mediated degradation.

For the somatic/developmental AID experiment, the TIR1 construct from the original *C. elegans* AID paper (Zhang et al 2015, PMID: 26552885) was used. This transgene drives TIR1 expression from an *eft-3* promoter. We have included this information in S1 Table.

4. Doesn't Fig. 3B show that N-tail homozygous F1 embryos have detectable level of FL by IF, and cannot be distinguished with het? This hinders me from believing that the difference in F1 and F2 N-tail homozygous is only due to the germ line.

It is correct that we cannot distinguish the different early F1 embryo genotypes based on the staining, since the heterozygous P0 mothers contribute a pool of the protein to the early embryos. We now state this in the corresponding figure legends. We assessed the persistence of the maternal CENP-A FL in the embryos, also in response to Reviewer 2 comment 9, and found that it is gradually diluted and remains detectable up to around 100-200-cell stage (new S2 Fig, lines 195-218). This narrows down the window in which the presence of CENP-A FL is essential from diplotene of the maternal germ line (where it first re-appears, Fig 1) to the ~200 cell stage embryos (where it disappears, S2 Fig). Together with the findings that the CENP-A ∆-tail protein is capable of functioning in mitosis (Fig 3), these observations leave two possible hypotheses: 1) mitosis in early embryogenesis is functionally distinct from mitosis post-gastrulation, and the former requires the N-terminal tail of CENP-A, whereas the latter does not. 2) The N-terminal tail is required before the first embryonic mitosis in the maternal germ line. It is difficult to envision why the CENP-A N-terminal tail would be essential for early, but not late embryonic chromosome segregation, and we therefore favor the second hypothesis, but we now also mention the first hypothesis in the text (lines 566-570).

To narrow down the essential time window for the CENP-A N-terminal tail experimentally, we expressed a pulse of CENP-A FL in adult F1 CENP-A ∆-tail homozygotes. This rescued the lethality in the F2 embryos, but only after a delay that is consistent with the FL protein being required before the onset of the first embryonic mitosis (new Fig 4D). We have revised the text to include these new results that support our hypothesis that the difference between the F1 and F2 homozygous embryos has its root in the germ line of the preceding generation (lines 286-301).

5. Although the authors mentioned that a strain containing a balanced CENP-A deletion produces homozygous CENP-A deletion that is embryonic lethal. This is a more fair comparison to imply the function of N-tail in embryos. As there is no ref, are the balancers the same. Have the authors performed IF of embryos to show the maternal load to germline and F1 embryos are really the same in N-tail homozygous and this CENP-A deletion?

We agree that the comparison of the CENP-A ∆-tail allele to the CENP-A KO allele is important. The balancer alleles are the same (hT2), as listed in S1 Table, and we now also state this in the text (lines 189-190).

As mentioned in response to comment 4, and also in response to Reviewer 2 comment 9, we assessed the persistence of the maternal CENP-A FL in the embryos, and found that it is gradually diluted and remains detectable up to around 100-200-cell stage. This is true for all investigated genotypes of the mothers: FL/FL, FL/∆-tail and FL/KO (new S2 Fig, lines 195-218).

6. Page12 line286

"Germ line of F1 CENP-A N-tail/N-tail animals, both CENP-A and KNL-2 show a punctate pattern, even though mitotis appears to be normal in these cells (S5 Fig)." The"punctate" pattern is apparent in the mitotic zone of germline (S5 Fig). However the CENP-A N-tail in diakinesis stage is not obviously different. How is mitotic accuracy in mitotic tip observed (Fig 4A)? How do the authors envision the punctate pattern (instead of on most of chromatin) affect centromere establishment/identity/localization?

We have revised S5 Fig to include full gonad pictures with zooms to show CENP-A and KNL-2 localisation in different cell stages in the germ line, also in response to Reviewer 1 comment 3 and Reviewer 2 comment 2. We are not sure how the punctate pattern in the mitotic zone of the germ line relates to the observed embryonic phenotypes, but we felt it was important to report it. It is visible in interphase nuclei, but not on condensed chromosomes, likely also explaining why CENP-A ∆-tail staining at diakinesis stage is not obviously different. The punctate patterns in the distal region do not appear to compromise centromere function, as F1 CENP-A ∆-tail homozygotes show normal development and germ line proliferation. Mitotic accuracy is observed by looking at metaphase plates and assessing bi-orientation, which appears normal. We clarify this in the text, lines 363-371.

7. Indeed, is it true that without the CENP-A N-tail interaction domain with KNL-2, the localization of CENP-A N-tail remains the same in diakinesis oocyte in Fig. 3B (with KNL-2) and in Fig. 5B (in KNL-2 depletion)? And what is the expected effect on centromere establishment/identity/localization?

We agree with the reviewer that the CENP-A ∆-tail staining during diakinesis looks similar in P0 (with CENP-A FL and viable progeny) and F1 animals (without CENP-A FL and inviable progeny). We hypothesize, however, that the genomic distribution is different in the two cases: In the P0 germ line, the interaction of CENP-A FL with KNL-2 (and LIN-53) is sufficient to set the correct centromere identity. In the F1 germ line, lack of interaction with KNL-2 results in loss of correct centromere identity and results in ectopic incorporation of CENP-A ∆-tail, mediated by LIN-53 (Fig 6C). This hypothesis predicts that the genomic distribution of CENP-A ∆-tail is different at diakinesis stage of P0 and F1 germ lines. However, with the imaging techniques that we use in this manuscript, we are unable to test this hypothesis, and development of the necessary germ cell genomics techniques are beyond the scope of this manuscript. We now discuss these points in the text, lines 371-374 and 514-539, also in response to Reviewer 2, comment 2.

8. Page 12 line 290 to 292

"Moreover, the removal of both CENP-A and KNL-2 at the mitosis-to-meiosis transition is delayed, and both proteins can be detected in part of the pachytene region of the germ line." The result or image cannot be found.

A similar point was brought up by Reviewer 2 in comment 2. We now show entire gonads of wt, P0 and F1 adults in S5A Fig. The CENP-A ∆-tail removal delay and its presence in pachytene cells is visible in about half of the germ lines analyzed.

9. Page 18 line 441 to 446

"We ruled out that the viability of the CENP-A ∆-tail F1 homozygotes solely depends on the presence of the maternal full-length protein. Instead, it is dependent on the presence of the full-length protein in the germ line of the preceding generation (P0). We therefore hypothesize that information about centromere identity rather than full-length CENP-A protein is transferred from the maternal germ line into the zygote (Fig 6B)." The authors hypothesize that it's centromere identity rather than the full-length CENP-A protein is inherited from germline to zygote, but the centromere identity in germline needs full-length CENP-A. It is unclear what could be the nature of the information of the identity that transmit? Based on Figure 6A, how does KNL-2 bring FL CENP-A to the correct genomic location in this establishment process?

Similarly, in Figure 6B what exactly is the meaning of "functional inheritance". In this model, F1 embryos still have residual materanla proteins also? If so, how can it be distinguished by the protein/RNA model? Can the authors at least propose what functional inheritance is mediated by? Histone modification?

We have revised the text in response to the comments above, and modified model Fig 7 (used to be Fig 6) to make our hypothesis more clear. In summary, we hypothesize that the interaction of CENP-A with KNL-2 through the CENP-A N-terminal tail is required to target CENP-A to the correct genomic regions to establish correct centromere identity. How KNL-2 identifies the correct genomic locations will be the subject of future studies. Once established, this centromere identity can then be maintained throughout development in the absence of the CENP-A N-terminal tail. The exact nature of correct centromere identity - which genomic regions need to be occupied - could not be determined in the context of this study, and alternative hypotheses such as a specific 3D organization of chromatin could also be envisioned. We show that maintenance of centromere identity is possible in the absence of the CENP-A N-terminal tail for one generation, likely involving an epigenetic maintenance mechanism that requires LIN-53. In the absence of the CENP-A N-terminal tail in the germ line of F1 animals, correct centromere identity cannot be established. We still observe LIN-53-dependent chromatin association of CENP-A ∆-tail, but we speculate that this chromatin association has an altered genomic distribution that does not result in a functional centromere in the F2 embryos. We have discussed these points in the text, lines 541-555

In Fig 7B we juxtaposed maternal contribution and maternal epigenetic inheritance. We have revised this figure to make this more clear, also in response to Reviewer 2, comment 6.

10. Ultimately, how can the authors attribute the F2 embryo chromosome missegregation to F1 germ line reestablishment, and not just no FL CENP-A in F2 embryos? The maternal load effect influence both germ line and the embryos produced. It may require a system that remove CENP-A in either the embryos or diakinesis, respectively. Is this feasible in the AID system developed here with early embryo re-expression/complementation?

We have addressed this in detail in response to comment 4. For the reasons explained there, our data strongly suggests that the essential role of the N-terminal tail is prior to the first mitotic division in embryos. However, we agree that the maternal contribution makes the determination of the precise time window difficult, and we now discuss this in the text, lines 566-570.

Minor:

11. Fig. 2B why the input KNL-2::OLLAS look so variable? While it is a 2-fold difference between 1 and 2, does the error bar suggests that they are not significantly different?

Fig. 2C blot seem to have bubble and distortion of band? No statistics?

A similar point was raised by Reviewer 1, comment 1. Repetition of the blots for the KNL-2 interaction with the H3.3 chimeras ended up increasing the variability of the results. We believe that this variability is the result of technical problems, but we were unable to solve these and to robustly quantify the interaction. We therefore decided to remove this panel from the figure. We note that the interaction of KNL-2 with the CENP-A N-terminal tail has recently also been described in a preprint from the Deasi lab (<https://www.biorxiv.org/content/10.1101/2020.12.28.424576v1>), which we now cite (citation 34).

We have added additional replicates to Fig 2B (used to be 2C).

12. p. 8 line 198 Please use CENP-A or hcp-3 consistently.

We have used the term CENP-A rather than HCP-3 to describe the centromere specific histone variant throughout the text in order to make it accessible to readers that are not familiar with *C. elegans* names. We have defined this on line 89. However, when we describe genotypes of the C. elegans strains used in this study, we cannot work around calling the *C. elegans* gene by its annotated name (*hcp-3*). This is the case in the methods section and in S1 Table. We have replaced HCP-3 with CENP-A in all other instances. We hope that it will be possible for the reader to understand the distinction.

13. Fig. 4A N-tail/N-tail F2 embryos KNL-2 cluster as big dots, but in Fig. 4B KNL-2 is diffused in prometaphase and metaphase, but have dots in anaphase. Can you explain the discrepancy? Can the authors propose why the mislocalized KNL-2 is at/with the microtubules?

The difference in KNL-2 distribution between Fig 5A and B (used to be 4A, B) stems from a mistake in assembling the panels that was also pointed out in Reviewer 2 comment 5, and for which we apologize. The ROD-1 movie still was inserted instead of the KNL-2 movie still in the ∆tail/∆tail panel. We have replaced the KNL-2 metaphase still, thereby resolving the discrepancy.

KNL-2 is detectable at low levels on MTs in wild type (S4A Fig), and we think that this effect becomes amplified when the interaction with CENP-A is lost. The relocalization to the spindle upon loss of CENP-A had already been observed in Maddox et al 2007 (PMID: 17339379; e.g. Video 5, Video 8). We now refer to this observation in lines 323-324.

14. p. 11 line 273 "We attribute these F2 embryonic phenotypes to the failure in establishing centromere identity in the germ line of F1 homozygotes (CENP-A N-tail/N-tail)." I think it is a premature attribution or conclusion.

We have made it clear that this is a proposal, rather than a firm conclusion (lines 343-345). Nevertheless, the F2 rescue experiments using a pulse of CENP-A FL expression in F1 adults that we now include (Fig 4D, S3 Fig) support this proposal.

15. Page 12 line 277 "CENP-A has previously been shown to be dispensable for meiosis" needs a citation. Ref 28?

We have changed the phrasing to “dispensable for chromosome segregation during meiosis in C. elegans” and added the missing reference (Monen et al 2005, PMID: 16273096, reference 28, line 348), also in response to Reviewer 2, comment 14.

16. Page 13 line 304 "depleting FL CENP-A in the proximal germ line did not lead to loss of KNL-2, as was reported in embryos" needs a citation for the embryo observation.

We have added the missing reference (Maddox et al 2007, PMID: 17339379, reference 30, line 386).

17. Page 13 line 308 The authors are discussing KNL-2-independent CENP-A N-tail localization, whereas the previous sentence is about CENP-A-independent KNL-2 localization. So both are indistinguishable from wild-type?

We have revised this part of the text, lines 406-409. In the germ line, KNL-2 localizes to chromatin independently of CENP-A. Deposition of CENP-A FL requires KNL-2. CENP-A ∆-tail, however, becomes uncoupled from KNL-2, and localizes the chromatin even in the absence of KNL-2.

18. Page 15 line 364 "However, non-mammalian KNL-2s often show cell cycle dynamics that are different……". Should it be "non-mammalian homologues of KNL-2"?

We have rephrased this sentence, lines 464-465.

19. Page 19 line 468 to 469 "RNAi experiments were performed using clones from the Ahringer library [80]". Although given the citation, the authors should at least mention that the RNAi experiments are performed by feeding RNAi. Non-worm people may be confused why the RNAi experiments were performed by using clones from a special library.

We have included the information as suggested, lines 612-614.

20. Page 20 line 484 to 485 "Images were obtained using a Leica DM5000 B microscope." Can the authors provide information on the use of objective and z-stack acquisition.

We have added the missing information in the updated methods section (lines 629-633). The images were acquired using 63x or 100x objectives. The DM5000 B is a widefield fluorescence/transmitted light microscope, so we cannot provide information about z-stack acquisition.

21. Page 20 line 489 to 490 "Young gravid adults were dissected on a 2% agarose pad and kept in egg buffer". Should it be "Young gravid adults were dissected in egg buffer and embryos were mounted on a freshly prepared 2% agarose pad"?

We have corrected the sentence as suggested, lines 637-639.

22. Have the authors looked at where the CENP-A N-terminus-H3.3 chimera localizes, and whether it complements some function of CENP-A?

The H3.3::CENP-A chimeras show a germline distribution that is similar to the one of CENP-A FL (absent in pachytene), but do not complement the CENP-A tail deletion.
