# Supplementary material for: Transgenerational inheritance of centromere identity requires the CENP-A N-terminal tail in the C. elegans maternal germ line
Source: PLoS Biol. 2021 Jul 6;19(7):e3000968. doi: 10.1371/journal.pbio.3000968 (PMC8259991; doi:10.1371/journal.pbio.3000968)
Supplement: Attachment — Submitted filename: Response_to_reviewers_round2.docx [file pbio.3000968.s019.docx]

**Point-by-point response to reviewers, revision round 2.**

We thank the reviewers again for their helpful comments. The line numbers refer to the final manuscript (without tracking changes).

Reviewers’ comments

Rev. 2:

The revised submission is much improved and the authors responded to the initial critiques in a positive way, including the addition of new data. The clarity and flow of the paper including data interpretation and presentation is better. The added experiments help better support the major claims in the paper. There are, however, a few minor points for the authors to consider in order to help clarify some of the new data:

1. Can the authors comment on why Figure 4A appears to show a lower percentage of the heterozygous male progeny compared to the homozygotes? Shouldn't it be 50-50 ratio as in 4B? Appears to be consistent for all broods which would argue against experimental variation.

We do not have an obvious explanation for why there is a bias towards homozygous offspring, as a 50-50 ration would be expected from mendelian segregation. It is possible that in this particular combination the segregation of the balanced allele is slightly disfavoured, which might also explain the higher than expected number of homozygous offspring in Fig 3A. We do not, however, observe this systematically, despite using the same balancer allele throughout our study.

2. The heat shock experiment is an elegant way of showing the stage specific assembly requirement in the maternal germline. The degradation of CENP-A obviously occurs outside of the time window (~4 hours) when de novo centromere establishment happens. Does the observed time frame of 4 hours coincide with diplotene to embryo developmental time as expected? This would be worth discussing and explaining further in the text (related to Figure 4D and S3).

We can only give an estimation, as we do not have the means to track individual germ cells for several hours. To estimate where in the germ line the precursors of the t4 embryos were at the time of the heat shock, we determined the number of embryos produced in the 4h after the heat shock. The average number of embryos in the uterus at t0 is 5.8. In the 4h after the heat shock, the worms lay on average 31.6 embryos. At t4, the average number of embryos in the uterus is 5.0. The number of embryos produced per gonadal arm is (31.6+5.0-5.8)/2=15.4. Using this number, we can count back and estimate that the (viable) embryos that are in the uterus at t4 were in diplotene stage at t0.

We have commented on this in the results (lines 312-316), discussion (lines 584-586) and methods sections (lines 733-741).

3. Figure 4C should have the generation (F2) also annotated directly in the panel, since 4B also has the same genotype but no particular generation.

We used the designations “F1” and “F2” for the generations after self-fertilization of the CENP-A FL/∆-tail “P0” heterozygotes. When crossing, this convention becomes difficult to apply. For example, in panel 4A, “P0” hermaphrodites are crossed to “F1” males, and the F1 male progeny of this cross are crossed back to “P0” hermaphrodites for multiple rounds. In this case the “F1” genotype is found in the prenatal and all the offspring generations. To avoid confusion, we decided not to apply the terms “P0”, “F1” and “F2” in the experiments involving crosses.

4. As the authors point out in lines 419-427, Lin53 plays a role in both maintenance and possibly establishment as it reduces the levels of both FL and delta tail CENP-A. But the authors could add that this reduction is more severe than that of KNL-2 depletion alone and hence provides further support for a role in 'maintenance'.

We find it difficult to directly compare the reduction of CENP-A upon depletion of KNL-2 and LIN-53, for technical reasons. The depletion was achieved by different methods, and the experiments were not done side by side. Moreover, the difference in the extent of depletion is not dramatic. We therefore prefer not to change the text.

Rev. 3:

This article examines the centromere protein A N-terminus function in centromere identity inheritance across generations and distinguishes that from CENP-A's mitotic function in mediating accurate chromosome segregation. In this revised version, the author used CENP-A KO to contrast with CENP-A N-tail deletion. They think that the relatively normal F1 is maintained by CENP-A N-tail deletion expressed in germline, as the counterparts with CENP-A KO don't exhibit the same behavior in F1. Also the AID system was used to show 2 points: 1/ the CENP-A N-del is able to support mitosis and development, 2/ the CENP-A N-del is unable to reload after pachytene (at some %?), but heat shock-mediated transient CENP-A FL expression (in diakinesis but not simply in embryos) can rescue the embryonic lethality. The manuscript has improved a lot with these additional experiments. However, there are multiple instances that the responses to reviewers' questions are much more clear and

straightforward (e.g. major comment 4 on the alternative hypothesis, and major comment 9 on the summary of the findings and the proposed epigenetic mechanism) than the wordings used in main text. The authors can consider using the justifications in text.

We thank the reviewer for this proposal, and we have replaced some of the text in the manuscript with text from the responses to comment 4 (lines 244-252) and 9 (lines 545-563). Incorporating the responses to comment 9 into the discussion created redundancies, and their removal required some rearrangement of the paragraphs in that part of the discussion.

Also for the minor suggestions below, while readers can probably guess what the authors mean, it will be better if the authors express more clearly what they mean explicitly.

Major:

1. Figure 4D. There is a 4-hour time frame between CENP-A FL expression and the rescue in early embryo segregation and development. What stage was it 4-hours before the first mitotic division? To non-worm audience, it is quite difficult to translate the 4-hour delay to developmental time frame. In addition to citing S3 Fig, could the authors explain this in the main text?

A similar point was brought up by Reviewer 2 in comment 2, and we have addressed it there. In short, we have determined that the average number of embryos produced per gonadal arm in the 4 h following heat shock is about 15, which would place the precursors of the viable embryos in diplotene stage at the time of the heat shock.

2. In Line 570, the author considered this delay strongly argues for a role of N-terminal tail in the germline rather than in the embryos. However, in S3 Fig, 0 h also has FL in diakinesis already. How long does it take from diakinesis to embryos? In other words, show for the "0 hr embryos", there is no FL x hrs earlier in the diakinesis. This is one of the most important added experiment and is worth describing more clearly.

To find out how long it takes from diakinesis to the (viable) “4h embryos”, we did the experiment described above. Determining the same for the “0h embryos” is not going to be informative. Since we heat shock adult animals, they have some embryos developing in their germline at any given point. The term “0h embryos” refers to the embryos already present within the uterus at the time of heat shock (i.e. when CENP-A FL expression from our construct is induced). These “0h embryos” are imaged right after the heat shock, and they therefore have developed from diakinetic oocytes that were never subjected to heat shock (the oocytes were fertilised before the heat shock pulse). We show in Fig 3 that there is no endogenous CENP-A FL protein in F1 ∆-tail/∆-tail adults, and we show in S3 Fig that there is no CENP-A FL protein produced from our inducible construct without heat shock pulse (“no hs”). Therefore, for the “0h embryos”, there is no CENP-A FL present in diakinesis at any given time before the heat shock.

Minor:

3. CENP-A is absent from centromere during pachytene, and indeed it is also absent in the paternal chromosomes before fertilization. They want to find out how CENP-A re-localizes to the centromere after CENP-A is removed from the chromatin in pachytene. They used the term "de novo establishment of centromeres" (Line32) to describe the centromere/CENP-A reappearance/re-establishment in meiosis I diplotene. The authors can consider using "re-" instead of "de novo".

We have changed it to re-establishment as suggested (line 32).

4. Figure 2B. + S1. Fig

In 2nd repeat of the IP triplicates, OLLAS (KNL-2) signal is weak for both tail and FL. However, in the main figure quantification, error bars in Figure 2B are small for KNL-2 enrichment. Specify the N, and if 2nd repeat in S1 Fig is excluded in quantification in Figure 2B, maybe it should be removed from the fig. (Previously commented as Q11, author responded: technical problem, redo and excluded that set of data from qualification.)

The second repeat is also included in the quantifications, as indicated in the legend to Figure 2B (N=3). Although the IP efficiency is weaker in the second repeat, the ratios remain similar to those of the other repeats.

The response to comment 11 referred to the IP of the different chimeras, where due to the technical problems we excluded the entire series of experiments, not just individual repeats.

5. Figure 3A

In the legend, "quantification of the viable offspring as a percentage of the expected hatchlings in the context of the balancer allele". Do the author mean that D-tail/balancer heterozygotes's hatching is set as 100%, and other genotypes are compared to this? If so, such normalization is good, but the figure Y-axis "% expected hatchings (balancer)" is very hard to understand. Maybe just say "% hatchings (normalized to balancer het)"?

We agree that understanding these quantifications is not straightforward. We have removed the “(balancer)” from the figure and have extended the description in the legend. 11/16 the offspring from the heterozygous P0 die because of aneuploidy or lethality caused by the balancer and were excluded from the scoring.

6. Figure 3B, 3D

Instead of * and explaining it in the legend the indistinguishable genotypes, the authors may as well put the two genotypes in the figure for clarity.

We have changed the figure and legend as suggested, also in response to comment 9.

7. Line 191, KO/KO "presumably receives the same dose of CENP-A FL....as those produced by CENP-A D-tail strain (Fig 3A)" It would be nice if this is actually shown by IF, to show that the maternal load (level) is not affected by the genetic background (or refer to S2B Fig here?). Line206-210 seems more relevant here.

The reviewer is correct, we indeed show by IF that the maternal load is not affected by the genetic background in S2B Fig and describe this result in the paragraph following this sentence. We removed the word “presumably” on line 191 and added “as described below'' (line 193).

8. Lin3 224, Figure 3C, can the authors briefly mention when is the soma-specific eft-3 promoter ON (or when FL is degraded) for the non-worm audience?

This comment refers to the developmental depletion of CENP-A using the degron system and a TIR1 transgene driven by an *eft-3* promoter. We rely on the original study that generated this strain (ref 36) and reported that the transgene is expressed in most or all somatic tissues throughout development, from embryos to adults. We have added this information to the results (lines224-225) and the methods section (lines 719-720).

9. Figure 3D, line 257

FL OLLAS staining in F1 embryos is from FL/D-tail zygotic? So indeed the 2 genotypes can be distinguished at this (later) stage of embryos? Then can show them separately instead of using *. But why is the signal not in all nuclei, but only in some?

Thank you for pointing out that the embryo in Fig 3D is indeed heterozygous, as it has zygotic expression of CENP-A FL. We have adapted the Figure and the legend.

The embryo is derived from an adult where CENP-A FL was depleted in the germ line, and upon this depletion all embryos show severe segregation defects and aneuploid cells, with proteins often showing mosaic expression patterns. We assume that this is caused by not all chromosomes being present in every cell.

10. Line 325, F2 embryos show "decondensation defects (Fig 5A). First, the defects should be "condensation defect"? Also, no condensation/decondensation assay was performed in this study. They can simply describe the shape of chromatin without inferring to the condensation process.

We were specifically asked to comment on the “observed defects in chromosome condensation” by Reviewer 1. But we agree that we did not perform a specific assay, and we have changed the sentence to “F2 embryos also show chromosomes that are less compacted (Fig 5A), which is similar to the condensation defects observed upon loss of CENP-A or KNL-2 [30].” (lines 340-342).

11. Figure 5

A has * as in Figure 3 B, D, but is not mentioned in legend.

B, C show maternal genotype FL/D-tail, but is it easier to annotate as in previous figures the two indistinguishable genotypes than to suddenly label maternal genotype?

We have replaced the maternal genotypes with the embryonic CENP-A phenotypes to make this figure consistent with the other figures.

12. Figure 6A

The bottom Auxin - or + should better reflect which AID protein is degraded (corresponding to the top label "KNL-2 depletion or CENP-A depletion"). Otherwise, it is very confusing to just look at the bottom graph.

We have now indicated which protein is depleted at the bottom of the graphs.

13. Line536. Typo: "maintans"

This typo has been corrected.

14. Line323. "S3A Fig" should be S4 Fig

The reference has been fixed (line 339).

15. Line 429-434. Consider rephrasing the summary of the findings "the CENP-A N-terminal tail is specifically required to re-establish correct centromere identity in......every generation" is more clear. Also "Once established, this centromere identity can then be inherited and maintained throughout development in the absence of the CENP-A N-terminal tail, but only......fails." to make it more clear.

We have changed the summary statement as suggested, with one addition. We think it is important to note that functional centromeres are maintained during development in the absence of the CENP-A N-terminal tail (lines 448-453).
